# MDA5 signaling induces type 1 IFN- and IL-1-dependent lung vascular permeability which protects mice from opportunistic fungal infection

**DOI:** 10.3389/fimmu.2022.931194

**Published:** 2022-07-28

**Authors:** Michael J. Davis, Rachel E. Martin, Giovana M. Pinheiro, Elizabeth S. Hoke, Shannon Moyer, Katrin D. Mayer-Barber, Yun C. Chang, Kyung J. Kwon-Chung

**Affiliations:** ^1^ Molecular Microbiology Section, Laboratory of Clinical Immunology and Microbiology, National Institute of Allergy and Infectious Diseases (NIAID), National Institutes of Health (NIH), Bethesda, MD, United States; ^2^ Inflammation and Innate Immunity Unit, Laboratory of Clinical Immunology and Microbiology, National Institute of Allergy and Infectious Diseases (NIAID), National Institutes of Health (NIH), Bethesda, MD, United States

**Keywords:** interferon, IL-1, *Cryptococcus*, edema, inflammation, lungs

## Abstract

Lungs balance threat from primary viral infection, secondary infection, and inflammatory damage. Severe pulmonary inflammation induces vascular permeability, edema, and organ dysfunction. We previously demonstrated that poly(I:C) (pICLC) induced type 1 interferon (t1IFN) protected mice from *Cryptococcus gattii* (Cg) *via* local iron restriction. Here we show pICLC increased serum protein and intravenously injected FITC-dextran in the lung airspace suggesting pICLC induces vascular permeability. Interestingly, pICLC induced a pro-inflammatory signature with significant expression of IL-1 and IL-6 which depended on MDA5 and t1IFN. Vascular permeability depended on MDA5, t1IFN, IL-1, and IL-6. T1IFN also induced MDA5 and other MDA5 signaling components suggesting that positive feedback contributes to t1IFN dependent expression of the pro-inflammatory signature. Vascular permeability, induced by pICLC or another compound, inhibited Cg by limiting iron. These data suggest that pICLC induces t1IFN which potentiates pICLC-MDA5 signaling increasing IL-1 and IL-6 resulting in leakage of antimicrobial serum factors into lung airspace. Thus, induced vascular permeability may act as an innate defense mechanism against opportunistic fungal infection, such as cryptococcosis, and may be exploited as a host-directed therapeutic target.

## Introduction

*Cryptococcus gattii* (Cg) is a fungal pathogen most recognized for a relatively large outbreak of cryptococcosis cases which occurred between 1997-2000 in the pacific northwest of north America while its sibling species, *Cryptococcus neoformans*, is mainly associated with deadly infections in AIDS patients world-wide (1–3). Although earlier reports suggested Cg to be a primary pathogen (4, 5), recent literature suggests that Cg is an opportunistic pathogen which often infects patients with uncharacterized pre-existing immune deficits (6–12). Unlike *C. neoformans*, Cg infections most often remain pulmonary in patients (8, 13) and experimental mouse models (14).

Previous work demonstrated that pharmacological stimulation of the host immune system protected mice from cryptococcosis (15, 16). These studies utilized Hiltonol (pICLC), a stabilized poly(I:C) derivative which signals through melanoma differentiation-associated protein 5 (MDA5), a cytosolic double stranded RNA sensor while other preparations of polyI:C signal through TLR3 (17). Like poly(I:C), pICLC is a molecular mimic of viral double stranded RNA. The best characterized product of MDA5 signaling is type 1 interferon (t1IFN), a family of cytokines initially identified as mediating antiviral effects, but more recent literature has shown more diverse effects (18). Nearly all mammalian cells express receptors for t1IFN and t1IFN signaling induces hundreds of responsive genes, many of unknown function. While pICLC-induced protection of mice from *C. neoformans* was dependent on pICLC-mediated induction of cellular immunity (16), these immune cells were all dispensable for pICLC-mediated protection from Cg. Instead pICLC-induced resistance to Cg was due to the induction of an iron restrictive-state caused by increased iron binding proteins in the lung airspace, the replicative niche of Cg (15). The presence of blood proteins in the lung airspace suggested the possibility of increased permeability in lung vasculature.

Studies show that increased lung vascular permeability is highly associated with inflammatory phenotypes, especially IL-1 (19) which induces permeability by regulated disruption of vascular adherens junctions. While the effects of IL-1 are the best characterized, other inflammatory factors may have important roles in regulating vascular permeability. Extensive lung vascular permeability is a defining feature of acute respiratory distress syndrome (ARDS), a devastating condition (20, 21). Though viral infection is a frequent cause of ARDS and poly(I:C) has been shown to exacerbate ventilator associated acute lung injury in mice (22, 23), type 1 interferon has not been shown to be directly associated with lung vascular permeability. While some of the molecular pathways and clinical consequences of lung vascular permeability have been explored, we know little about why mammals induce vascular leakage in response to inflammatory signals. We questioned whether inflammation associated vascular permeability may benefit the host in certain circumstances.

Our previous data showing induction of several blood proteins in the lung airspace led us to hypothesize that serum factors were being delivered by pICLC-induced permeability in the lung vasculature. Our data show that pICLC induces measurable lung vascular permeability dependent on MDA5, t1IFN, IL-1, and IL-6. This vascular leakage limited fungal Cg pulmonary growth whether induced by pICLC or by a known inducer of vascular leakage, c48/80. These data provide insights into the contribution of MDA5 and t1IFN signaling pathways in the induction of lung edema and the basis for the linkage of inflammation and the induction of vascular permeability.

## Materials and methods

### Mice

Wildtype C57BL/6 mice and IL-6 -/- mice were purchased from The Jackson Laboratory (Bar Harbor, ME). MDA-5-/-, IFNar1-/-, and IL-1r1 -/- mouse strains were all purchased under the NIAID supply agreement with Taconic. All mice were females aged 8-12 weeks at the start of experiments.

### Ethics statement

The Institutional Animal Care and Use Committee of the National Institute of Allergy and Infectious Diseases approved all animal studies (approval no. LCIM-5E). Studies were performed in accordance with the recommendations of the Guide for the Care and Use of Laboratory Animals of the National Institutes of Health.

### Intrapharyngeal aspiration dosing and compounds

pICLC, Cg, Compound 48/80, and iron chloride were dosed to mice using intrapharyngeal aspiration as previously (15). PICLC (Hiltonal) was a generous gift of Andres Salazar (Oncovir, Inc; Washington DC). PICLC was diluted in sterile PBS and dosed at 5 µg in 20 µL at the indicated time points. Compound 48/80 (Sigma-Aldrich) was diluted in sterile PBS and dosed at indicated amounts. Iron (III) Chloride (Sigma-Aldrich) was prepared as previously (15). Briefly, Iron chloride solutions were sterile filtered, neutralized, and diluted in PBS and dosed at 6.25 µg per dose in 20 µL.

### *Cryptococcus* strains and culture

*Cryptococcus gattii* strain R265 was maintained as frozen glycerol stocks as previously (15). At the start of each experiment, a fresh frozen aliquot was thawed and cultured overnight in YPD (MP Biomedicals, Santa Ana, CA) at 30°C. Yeast cells were harvested by centrifugation, counted by hemocytometer, and diluted to 2.5x10^5^ yeast per mL in sterile PBS, resulting in 5000 yeast per mouse in 20 µL.

### Mouse sample collection and analysis

See **Supplemental Figure 1** for dosing and sampling schematic. At indicated timepoints, terminal retro-orbital bleeds for serum collection were performed under deep isoflurane anesthesia. Then mice were immediately sacrificed using CO_2_, their tracheas exposed, and bronchoalveolar lavage (BAL) collected using 1 mL of sterile PBS containing EDTA-free protease inhibitor (Roche). Lavage samples were cleared by centrifugation and supernatants stored at -80°C. Lavage samples were monitored for blood contamination and contaminated samples were excluded from further analysis. Note that preliminary experiments demonstrated significant difficulty in collecting sufficiently pure lavage samples from Cg infected mice at time points longer than 7 days post infection.

BAL and serum samples were analyzed for transferrin, albumin, complement c3, ferritin, and lipocalin-2 protein levels by ELISA kits purchased from Immunology Consultants Laboratory, Inc (Portland, OR). Total protein levels were measured by BCA Protein assay kit (Pierce from Thermo-Fisher). Cytokine and chemokine levels were measured by custom Procartaplex Luminex panel (Thermo-Fisher). ELISAs, BCA assays, and Luminex analysis were all performed by manufacturer’s specification.

For Fold change heat maps of cytokine and chemokine data, fold change calculations were performed in Excel (Microsoft) and statistical analysis in GarphPad Prism (San Diego, CA). Fold changes for each condition were averaged and those with no statistical difference from control (wildtype – PBS) were set to black. Heat map image files were generated using the Morpheus software (Broad Institute; https://software.broadinstitute.org/morpheus).

### Dye tracer vascular permeability assay

**Figure 1:**
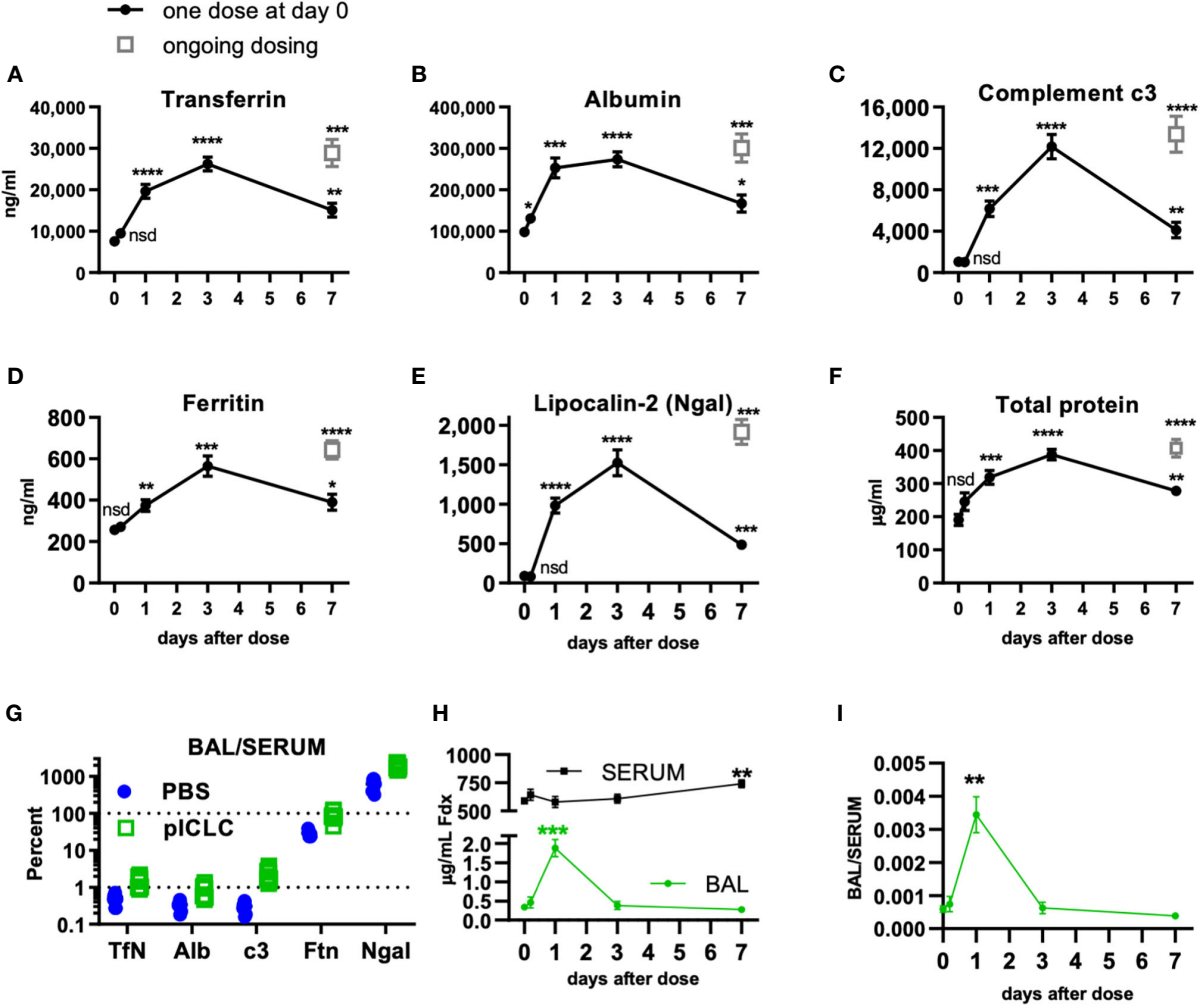
Locally dosing mice with pICLC results in accumulation of serum proteins in the lung airspace. C57BL6 mice were dosed with 5 µg of pICLC by intrapharyngeal aspiration either once at day 0 (black closed circles with lines) or repeatedly (grey open boxes; ongoing dosing) on days 0, 3, and 6. **(A–F)** BAL samples were acquired at indicated time points. See **Supplemental Figures 1A, B** for dosing and sampling schematic. Analytes from BAL samples were measured using ELISA **(A–E)** or total protein assay **(F)**. N = 10-14 mice per timepoint per group combined from 3 independent experiments. Statistics analyzed by one way Brown-Forsythe ANOVA and subsequent multiple comparison corrected t-tests compared to PBS control. **(G)** Paired serum and BAL samples were acquired 3 days following pICLC or PBS dosing and analyzed by ELISA. BAL analyte concentrations were divided by serum concentrations from the same animals. N = 9-10 mice per group combined from 2 independent experiments. **(H, I)** Mice were dosed once with pICLC or PBS as in A-F then intravenously injected with 4 µg FITC-dextran (Fdx). Fdx levels in paired BAL and serum samples were measured 3 hours after Fdx injection. N = 8-10 mice per timepoint per group combined from 2 independent experiments. **** indicates p < 0.0001, *** indicates p < 0.0002, ** indicates p < 0.0021, * indicates p < 0.0332, nsd indicates not statistically different.

Mice were pICLC or PBS dosed as indicated. Mice were then intravenously injected with (4 µg in 200 µL PBS) FITC-dextran 70 kD average molecular weight (Sigma-Aldrich) then after about 3 hours serum and BALs were collected as described above. Fluorescein fluorescence was then measured in the BAL, 1:100 diluted serum samples, and Fdx standards of known concentration using a Synergy H1 plate reader (BioTek) in fluorescence mode with 485 nm excitation and 528 nm emission. Sample Fdx concentrations, in µg/mL, were calculated using standard curve interpolation. BAL/Serum values in **Figure 1I** are BAL µg/mL divided by serum µg/mL values from the same mouse.

### Measurement of lung fungal load

Mice were euthanized and lungs excised at the indicated timepoints. Lungs were homogenized in PBS containing EDTA-free protease inhibitor (Roche). Colony forming units in each lung were then calculated using colony forming unit dilution plating.

### Gene expression analysis

At indicated time points mouse lungs were harvested and homogenized in TRIzol (Thermo-Fisher). RNA was purified from samples using TRIzol Plus RNA Purification Kit with Phasemaker Tubes (Thermo-Fisher). RNA concentration was measured using a Nanodrop Spectrophotometer ND-1000 (Nanodrop Technologies), DNAse treated using DNA-free DNA removal kit (Thermo-Fisher), and reverse transcribed using TaqMan Reverse Transcription Reagents (Thermo-Fisher) all according to manufacturer’s instructions. Taqman real time PCR was performed using TaqMan Fast Advanced Master mix (Thermo-Fisher), a QuantStudio 3 pPCR machine (Applied Biosystems by Thermo), and the following TaqMan assay probes from Thermo-Fisher: ifih1 (MDA5) Mm00459183_m1, isg15 Mm01705338_s1, ifnb1 Mm00439552_s1, il1b Mm00434228_m1, il10: Mm00439614_m1, ch25h: Mm00515486_s1, gapdh: Mm99999915_g1. Gene expression fold change was calculated using delta delta Ct analysis using gapdh as control.

### Statistics

ANOVA and subsequent pairwise statistical analysis were performed using Graphpad Prism (San Diego, CA) as indicated in figure legends. All graphs indicate means with SEM error bars.

## Results

### pICLC-induces alterations in lung vascular barrier function

Previous data demonstrated that transferrin and other serum iron-binding proteins were elevated in lung airspace samples from pICLC-dosed mice (15) leading us to hypothesize that pICLC induces permeability in the lung vasculature. To convincingly demonstrate lung vascular permeability in rodent models, the American Thoracic Society (ATS) suggests presenting studies of three of the four common permeability phenotypes (24): 1) alterations in alveolar barrier function, 2) histological evidence of permeability, 3) induction of an inflammatory response, and 4) physiological dysfunction.

To further demonstrate pICLC-induced alterations in alveolar barrier function, mice were dosed once with pICLC or PBS by intrapharyngeal aspiration on day 0 and bronchoalveolar lavage (BAL) samples were acquired at various times thereafter. BAL samples were also acquired after 7 days of ongoing pICLC-dosing (**Supplemental Figure 1**). Transferrin (Tfn), ferritin (Ftn), albumin (Alb), lipocalin-2 (Ngal), and complement c3 were all induced into the lung airspace by a single pICLC dose and ongoing dosing (**Figures 1A–E**). Additionally, total protein levels were also elevated in BAL samples from pICLC-dosed mice (**Figure 1F**). These parameters all displayed similar kinetics with increased expression one day after pICLC-dosing, peaking at 3 days, then returned to nearly baseline after 7 days. The presence of serum proteins and the elevation in total protein in BAL samples is consistent with pICLC-induced alteration in lung vascular permeability.

Interestingly, two distinct patterns emerged when analyte BAL/serum analyte concentrations were compared. Tfn, Alb, and c3 all show similar and relatively moderate BAL/serum ratios while Ftn and Ngal had much higher BAL to serum ratios (**Figure 1G**), suggesting that Tfn, Alb, and c3 are delivered to lung airspace by vascular permeability while Ftn and Ngal seem to be delivered to airspaces by other mechanisms. Thus, we selected Tfn and Alb as markers for permeability in further experiments.

Finally, to definitively demonstrate a pICLC-induced alteration in lung vascular barrier function pICLC-dosed or control mice were injected intravenously with FITC-dextran and the concentration of FITC-dextran measured from serum and BAL samples. FITC-dextran concentration (**Figure 1H**) and FITC-dextran BAL/SERUM ratios (**Figure 1I**) were elevated in BAL samples 1 day after pICLC dosing then returned to baseline. Collectively, the pICLC-induced leakage of lung serum proteins and tracer molecules into lung airspaces demonstrates pICLC-induced alteration in lung vascular barrier function.

### pICLC-induces histological evidence of lung vascular permeability

Moderate vascular permeability increases result from endothelial cell junctions loosening, resulting in capillary vessels dilation. Since alveolar walls themselves are not much larger than their capillaries, increased vascular permeability often presents histologically as thickened alveolar walls (24). Histological lung samples were acquired one or three days after dosing with PBS, pICLC, or compound 48/80 (C48/80), a known inducer of lung vascular leakage (25, 26). PBS control samples showed very thin alveolar walls (**Supplemental Figure 2A** left image) while lung sections from pICLC dosed mice displayed modestly thicker walls (**Supplemental Figures 2B, C**). High-dose C48/80 induced more dramatic alveolar wall thickening (**Supplemental Figures 2D, E**) and more lung vascular permeability (see **Figures 7A–C** below). Some modestly increased cellularity was also observed in pICLC dosed sections (**Supplemental Figures 2B, C**) with higher cellularity in C48/80 dosed mice (**Supplemental Figures 2D, E**). Also note that obvious changes in fibrotic markers were not observed in pICLC-treated lung sections (**Supplemental Figure 2** left panels). To confirm the increased cellularity in pICLC-dosed mice, neutrophils and monocytic cells were enumerated by flow cytometry showing a recruitment kinetic in pICLC-dosed lungs similar to Tfn (**Supplemental Figure 3**). While these cells are indicative of inflammatory levels, these recruited cells were previously shown to be dispensable in this model (15). Overall, pICLC induced some alveolar wall thickening and leukocyte recruitment but without more drastic pathology seen in other models of alveolar damage (24).

### pICLC-induces robust pro-inflammatory signals

**Figure 2:**
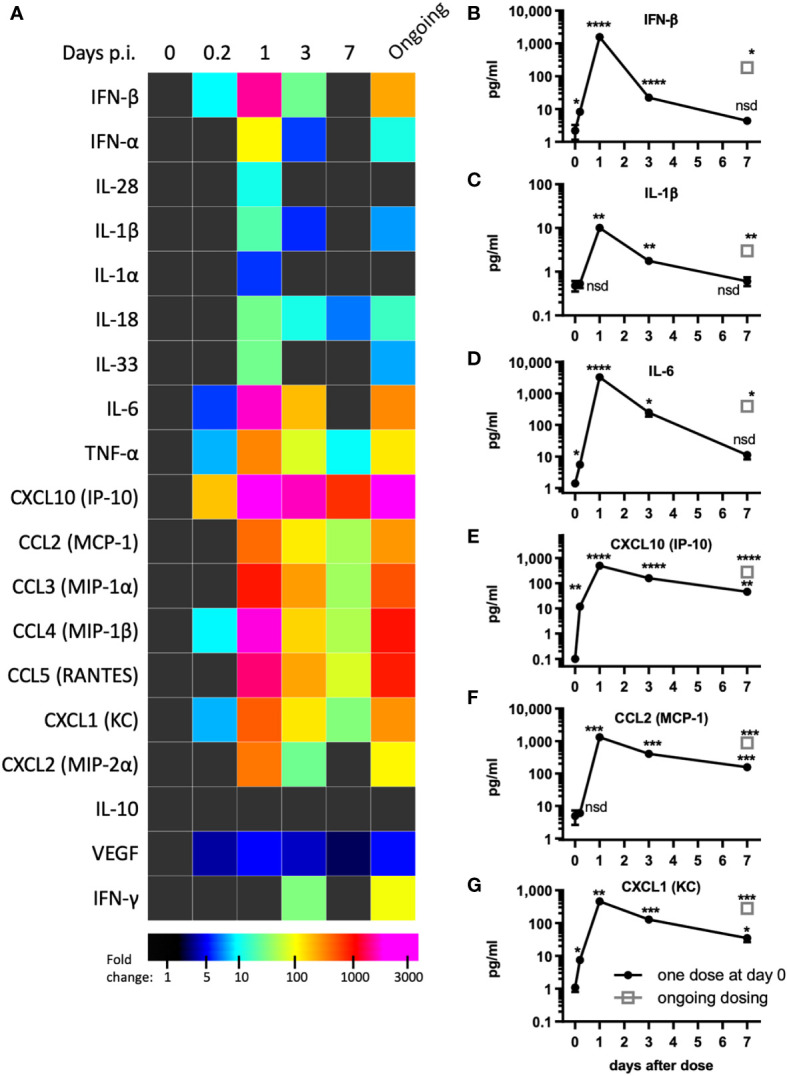
pICLC induces type 1 IFN and inflammatory cytokines and chemokines. Mice were dosed with pICLC or control as in **Figures 1A–F** and cytokine and chemokine levels were measured in BAL samples by bead-based assay. **(A)** Fold change values are displayed using a heatmap. **(B–G)** data from select analytes are graphed as in **(A-F)**. N = 10 mice per timepoint per group combined from 2 independent experiments. Statistics analyzed by one way Brown-Forsythe ANOVA and subsequent multiple comparison corrected t-tests compared to PBS control. **** indicates p < 0.0001, *** indicates p < 0.0002, ** indicates p < 0.0021, * indicates p < 0.0332, nsd indicates not statistically different.

Tissue damage and inflammation are the best characterized drivers of vascular permeability (19, 24) however, evidence of tissue damage was absent in pICLC-dosed lungs suggesting pro-inflammatory mediators may underlie pICLC-induced vascular permeability. Poly(I:C) and pICLC are routinely used to induce t1IFN but inflammatory cascade induction by poly(I:C) is substantially less characterized and probably context dependent. Thus, a variety of cytokines and chemokines were measured in pICLC and PBS dosed mice. As expected, pICLC induced robust IFN-β/α responses (**Figures 2A, B** and **Supplemental Figure 4**) which were detectable above background after 2 hours, peaked after one day, then returned to baseline after seven days. CXCL10 is a t1IFN-induced chemokine (27) and its expression was also quite consistent with t1IFN (**Figures 2A, E**). In contrast, IL-28 (IFN-λ) was barely observed (**Figure 2A** and **Supplemental Figure 4**).

PICLC also induced a robust pro-inflammatory response. The IL-1 family cytokines IL-1β, IL-1α, IL-18, and IL-33 were all induced by pICLC. Of this family, IL-1β and IL-18 were particularly induced (**Figures 2A, C** and **Supplemental Figure 4**). IL-6 (**Figures 2A, D**), TNF-α (**Figure 2A** and **Supplemental Figure 4**) and inflammatory responsive chemokines were also highly induced (**Figures 2A, F, G** and **Supplemental Figure 4**). In general, these cytokines showed similar kinetics to t1IFN. Thus, pICLC induces a substantial pro-inflammatory response in addition to the well characterized t1IFN response.

Taken together these data demonstrate that pICLC induces inflammatory mediators, alterations in vascular barrier function, and histological evidence of alveolar wall thickening which meet the guidelines for vascular permeability set by the ATS (24). We next considered the physiological relevance of this conclusion and the pathways involved.

### MDA5 signaling induces t1IFN, inflammatory mediators, and vascular permeability

**Figure 3:**
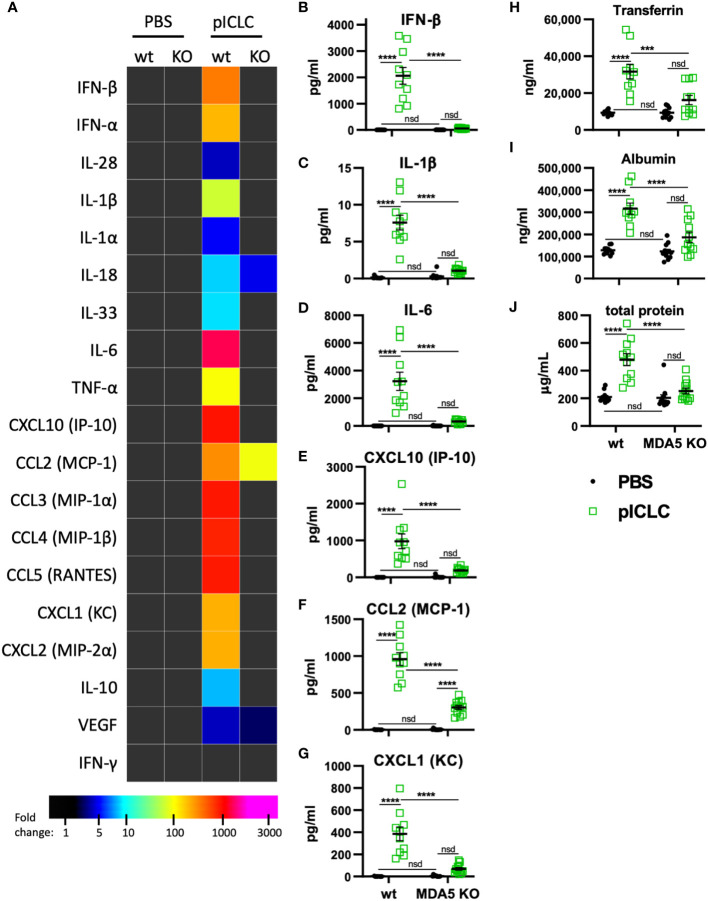
pICLC-induced cytokine and permeability responses are MDA5 dependent. MDA5 KO (KO) and C57BL6 control mice (wt) were dosed with PBS (black symbols) or pICLC (green) and BAL samples acquired 1 day **(A–G)** or 3 days **(H-J)** later. See **Supplemental Figure 1C** for dosing and sampling schematic. **(A–G)** Cytokine and chemokine levels measured in BAL samples by bead-based assay as in **Figure 2** and displayed by heatmap **(A)** or graphs of selected analytes **(B–G)**. Markers of vascular permeability were measured by ELISA **(H, I)** or BCA assay **(J)** as in **Figure 1**. For **(A–J)**, N = 10-12 mice per group combined from 2 independent experiments. Statistics analyzed by two-way ANOVA and subsequent Tukey multiple comparison tests. **** indicates p < 0.0001, *** indicates p < 0.0002, nsd indicates not statistically different.

Previous data showed that pICLC-mediated protection of mice from cryptococci depended entirely on MDA5 (15, 16), however MDA5 signaling has not been shown to mediate vascular permeability. To evaluate MDA5’s role in vascular permeability and cytokine induction, we dosed MDA5-deficient (MDA5 KO) and control mice with pICLC and measured cytokines, chemokines, and vascular permeability markers. IFN-α/β and the IFN responsive chemokine, CXCL10, were all highly induced by pICLC in wildtype mice but not MDA5 KO mice (**Figures 3A, B, E** and **Supplemental Figure 5**) confirming that t1IFN expression is MDA5 dependent. Notably, pro-inflammatory cytokine and chemokine expression, including IL-1 and IL-6, was also dependent on MDA5 (**Figures 3C–G** and **Supplemental Figure 5**). Tfn, Alb, and total protein in BAL were elevated in pICLC-dosed wildtype animals while they were at baseline in pICLC-dosed MDA5 KO mice (**Figures 3H–J**). Together these data show that t1IFN, pro-inflammatory mediators, and lung vascular permeability were entirely dependent on MDA5, demonstrating a novel role for MDA5 signaling in lung vascular permeability.

### pICLC- and MDA5-induced vascular permeability and pro-inflammatory mediators depend on t1IFN

**Figure 4:**
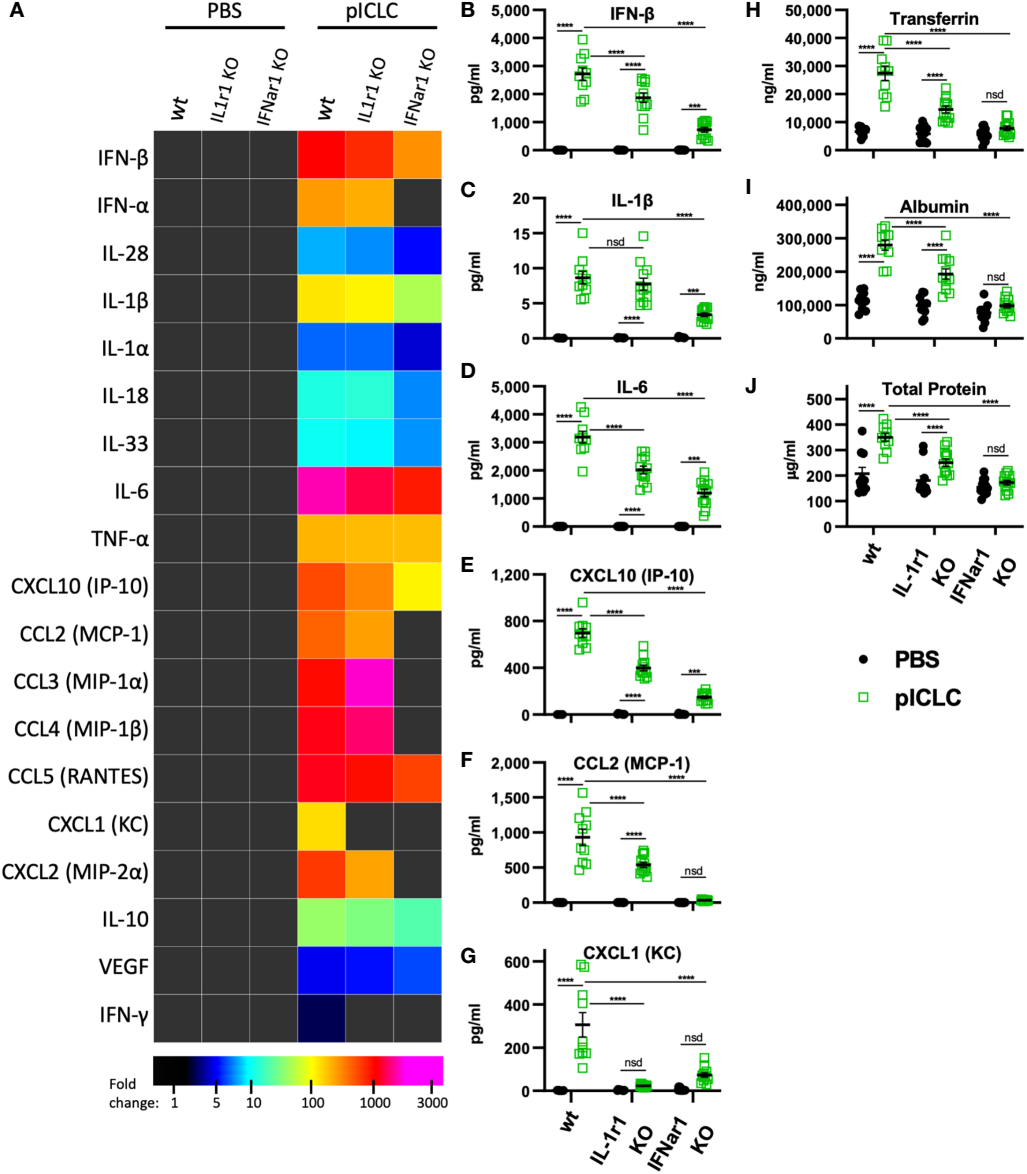
pICLC-induced vascular permeability depends on both t1IFN and IL-1, while the pICLC-induced cytokine response depends mostly on t1IFN. IFNar1 KO, IL-1r1 KO, and C57BL6 control mice (wt) were dosed with PBS (black symbols) or pICLC (green) and BAL samples acquired 1 day **(A–G)** or 3 days **(H–J)** later. See **Supplemental Figure 1C** for dosing and sampling schematic. Cytokine and vascular permeability analytes measured and displayed as in previous experiments. For **(A–J)**, N = 10-12 mice per group combined from 2 independent experiments. Statistics analyzed by two-way ANOVA and subsequent Tukey multiple comparison tests. **** indicates p < 0.0001, *** indicates p < 0.0002, nsd indicates not statistically different.

While several inflammatory cytokine pathways have been shown to induce lung vascular permeability, t1IFN has not been shown to impact this process. Thus, we tested pICLC-responses in mice genetically deficient in the common t1IFN receptor (IFNar1 KO). IFNar1 KO and wildtype control mice were pICLC dosed and cytokines and lung vascular permeability markers measured. IFN-α/β and CXCL10 expression were markedly reduced in pICLC-dosed IFNar1 KO compared to control mice (**Figures 4A, B** and **Supplemental Figure 6**), consistent with previous reports that t1IFN exhibits positive feedback by inducing its own expression (28–30). Expression of IL-1β, IL-6, CCL2 and other pro-inflammatory factors was significantly reduced in pICLC-dosed IFNar1 KO mice compared to control mice (**Figures 4A–G** and **Supplemental Figure 6**). Together, these data indicate that the pICLC-MDA5 induction of IL-1 and other pro-inflammatory factors was largely dependent on t1IFN signaling.

While Tfn, Alb, and total protein levels were strongly elevated in pICLC-dosed wildtype mice, all lung vascular permeability markers were at baseline in IFNar1 KO mice dosed with pICLC (**Figures 4H–J**) or Cg infected IFNar1 KO mice repeatedly dosed with pICLC (**Figure 6A**). Thus, pICLC-induced vascular permeability is entirely t1IFN dependent.

### The pro-inflammatory pICLC-MDA5 response is downstream of t1IFN

To probe the role of IL-1 signaling in these processes, we utilized mice deficient in the common IL-1 receptor (IL-1r1 KO). Interestingly, the cytokine response to pICLC was only slightly reduced in IL-1r1 KO mice. Most measured pro-inflammatory cytokines and chemokines, including IL-1 and IL-6, showed only minor reductions in pICLC-dosed IL-1r1 KO mice compared to wildtype mice (**Figures 4A–F**). In contrast, the neutrophil attractant chemokines CXCL1 and CXCL2 were eliminated or significantly reduced respectively, in pICLC-dosed IL-1r1 KO mice compared to similarly dosed wildtype (**Figures 4A, G** and **Supplemental Figure 6**). IFN-α, IFN-β, and CXCL10 expression were modestly reduced in pICLC-dosed IL-1r1 KO mice (**Figures 4A, B, E** and **Supplemental Figure 6**) suggesting t1IFN is only minorly dependent on IL-1 signaling. Overall, the t1IFN and pro-inflammatory pICLC responses were only slightly dependent on IL-1 signaling. Since IL-1 was highly dependent on IFNar1 but t1IFN was largely not dependent on IL-1r1, these data indicate that IL-1 and other pro-inflammatory factors are downstream of t1IFN in this model.

### pICLC induced vascular permeability depends on IL-1 and IL-6 signaling

IL-1, one of the pICLC-induced proinflammatory mediators, has a relatively established role in inducing vascular permeability (19). Thus, we hypothesized that IL-1 induced by pICLC was mediating the observed lung vascular permeability. Tfn, Alb and total protein were significantly reduced in BALs from IL-1r1 KO mice compared to wildtype in single pICLC-dosed mice (**Figures 4H-J**) and in Cg infected IL-1r1 KO mice repeatedly pICLC dosed mice (**Figure 6B**). Thus, pICLC-induced vascular permeability is strongly dependent on IL-1 signaling.

**Figure 5:**
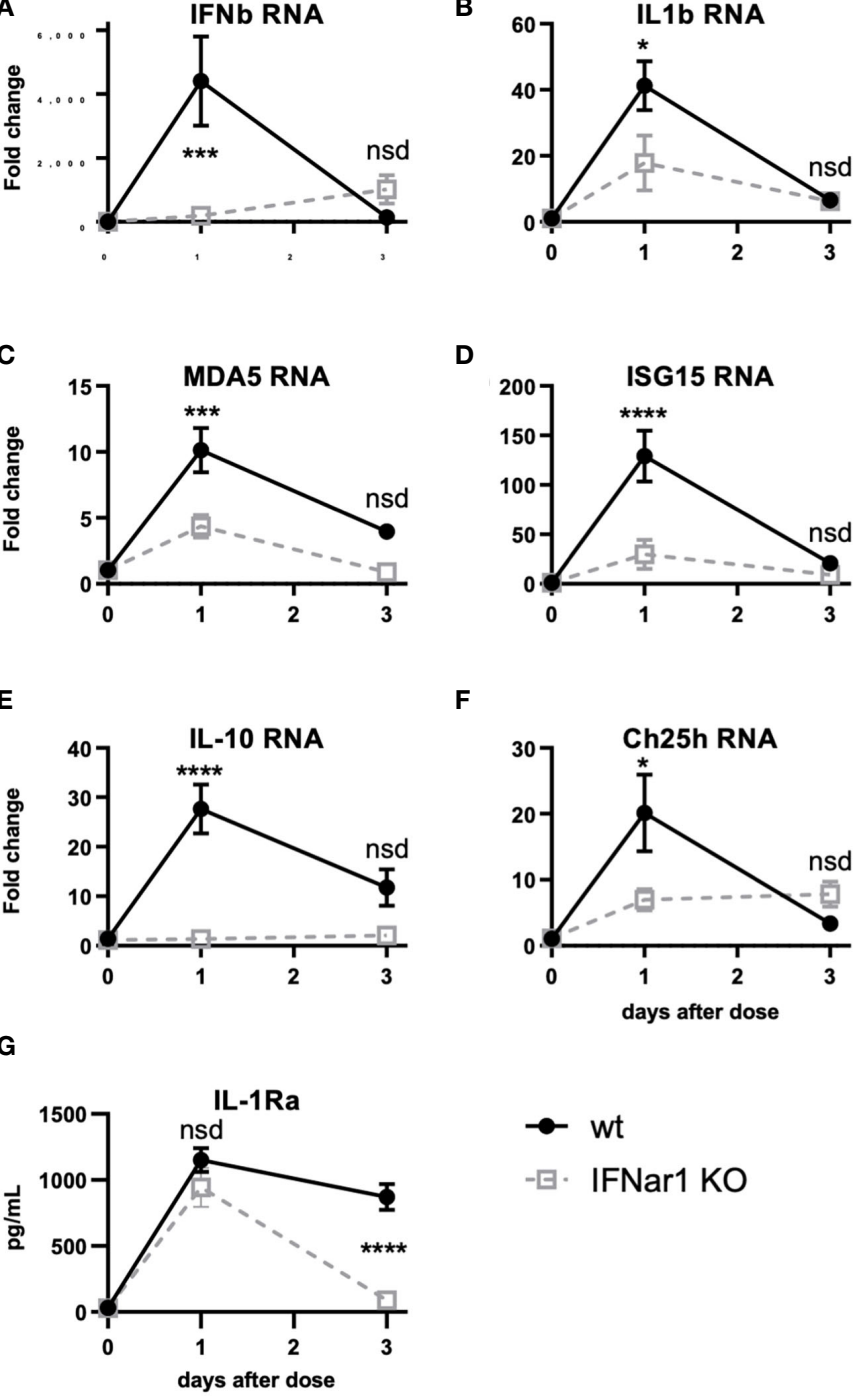
t1IFN induces expression of MDA5 and ISG15 as well as downstream genes, including IL-1. IFNar1 KO and C57BL6 control mice (wt) were dosed with PBS (time point 0) or pICLC and lung mRNA **(A-F)** or lung homogenate **(G)** harvested 1 or 3 days later. **(A-F)** mRNA levels for indicated genes were measured by qPCR. **(G)** IL-1Ra levels were measured by ELISA. N = 8 mice per group combined from 2 independent experiments. Statistics analyzed by two way ANOVA and subsequent Tukey multiple comparison tests. **** indicates p < 0.0001, *** indicates p < 0.0002, * indicates p < 0.0332, nsd indicates not statistically different.

**Figure 6:**
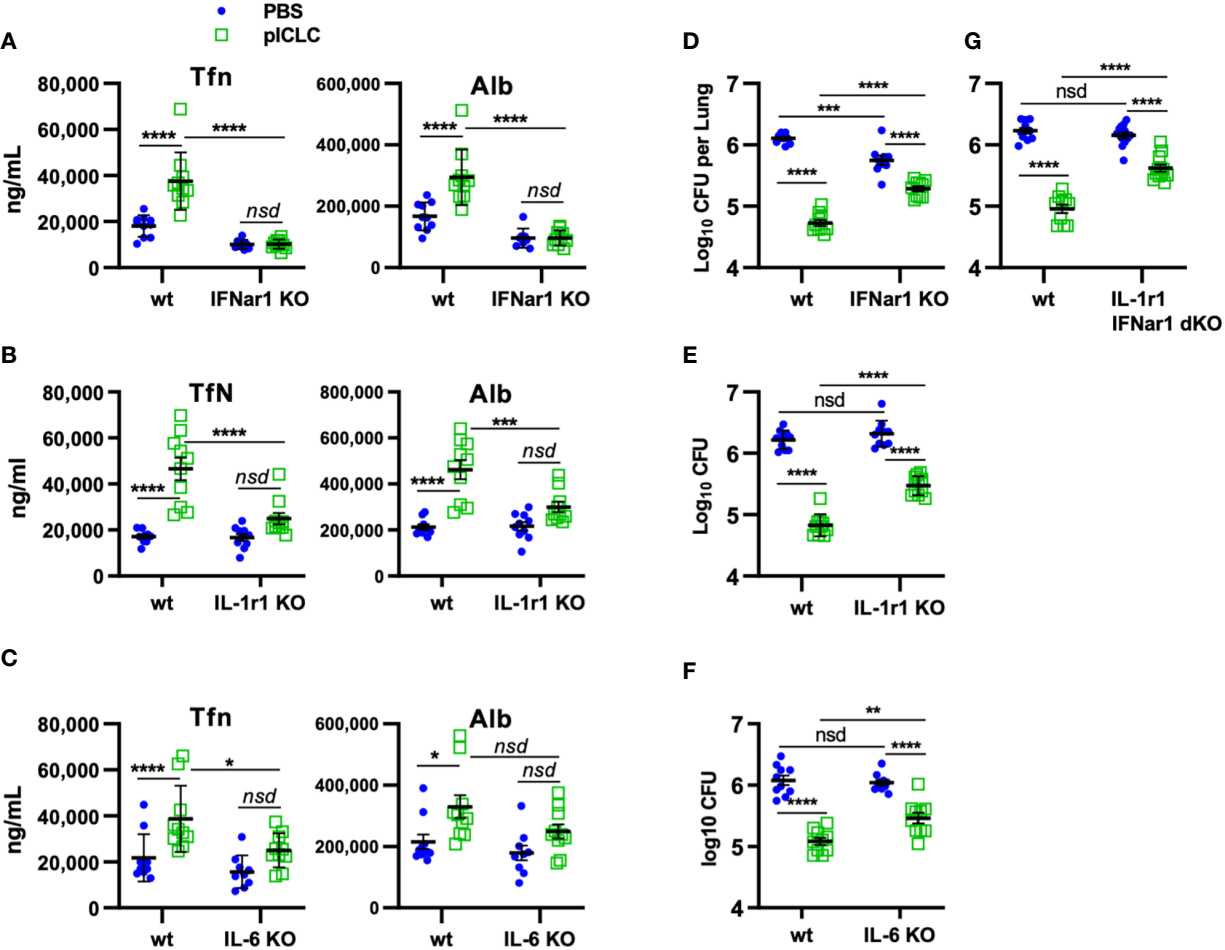
pICLC-mediated protection from opportunistic *C. gattii* infection depends on t1IFN, IL-1, and IL-6. IFNar1 KO, IL-1r1 KO, IL-6 KO, IFNar1 and IL-1r1 double KO, and C57BL6 control mice (wt) were infected with *Cryptococcus gattii* (5000 cells) and dosed with PBS (blue symbols) or pICLC (green). 7 days post infection BAL samples were analyzed by ELISA for markers of vascular permeability **(A–C)** and lung homogenates were analyzed for fungal load **(D–G)**. See **Supplemental Figure 1D** for dosing and sampling schematic. N = 8-10 mice per group combined from 2 independent experiments. Statistics analyzed by two-way ANOVA and subsequent Tukey multiple comparison tests. **** indicates p < 0.0001, *** indicates p < 0.0002, ** indicates p < 0.0021, * indicates p < 0.0332, nsd indicates not statistically different.

IL-6 has also been shown to mediate lung vascular permeability in some viral models (31, 32) and was also induced by pICLC and t1IFN (**Figure 5D**). Thus, we hypothesized that IL-6 may contribute to pICLC-induced lung vascular permeability. Cg infected pICLC-dosed IL-6-deficent mice showed marked reduction in BAL Tfn and Alb compared to wildtype mice (**Figure 6C**). Thus, pICLC-induced lung vascular permeability depends on both IL-1 and IL-6 while the expression of these cytokines depends on t1IFN signaling. Taken together these data suggest that pICLC, MDA5, and t1IFN induce a multifactor pro-inflammatory response which stimulates lung vascular permeability.

### T1IFN induces pro-inflammatory response by inducing MDA5 expression positive feedback

**Figure 4** shows that t1IFN is upstream of IL-1 and IL-6 following pICLC-MDA5 stimulation. However other data show that t1IFN and IL-1 have a complicated signaling interplay and, in some contexts, a cross inhibitory relationship (33–38). Also, t1IFN stimulates positive feedback mechanisms which potentiate its own expression [**Figures 4A, B** and (28–30)]. We hypothesized that t1IFN induces IL-1 and other pro-inflammatory factors by enhancing the MDA5 signaling pathway forming a positive feedback loop. Thus, lung RNA expression was measured in IFNar1 KO and wildtype mice dosed with pICLC or PBS. Consistent with protein data (**Figures 4B** and **C**), IFNb and IL1b gene expression was pICLC-induced and IFNar1 dependent (**Figures 5A, B**). MDA5 (**Figure 5C**) and ISG15 (**Figure 5D**) also showed IFNar1-dependent pICLC-mediated induction. ISG15 has been shown to increase the signaling efficiency and flux through the MDA5 pathway (28). These data support that pICLC-induced t1IFN induces factors enhancing MDA5 signaling. Consistent with previous findings by other groups (36, 37, 39), pICLC mediated IFNar1-dependent expression of IL-10 mRNA (**Figure 5E**), Ch25h mRNA (**Figure 5F**), and IL-1Ra protein (**Figure 5G**).

### pICLC-induced protection of mice from Cg depends on IFNar1, IL-1, and IL-6

Our previous work demonstrated that pICLC-mediated protection from *Cg* was mediated by iron scarcity due to a pICLC-induced influx of iron-binding proteins (15). The data above demonstrate that lung vascular permeability induced by pICLC-, MDA5-, and t1IFN-dependent IL-1 and IL-6 delivers serum iron proteins to lung airspaces. This predicts that pICLC protection from Cg also depends on these cytokines. To probe the role of these cytokines in the pICLC-induced protection from Cg, genetically deficient or wildtype animals were Cg infected and treated with pICLC or PBS. Consistent with previous data, IFNar1 KO mice dosed with pICLC lose substantial Cg inhibition (**Figure 6D**). Similarly, IL-1r1 KO mice and IL-6 KO mice dosed with pICLC also lose substantial Cg inhibition (**Figures 6E, F**). Data in **Figure 4** suggest that t1IFN, IL-1 and IL-6 are all functioning in the same pathway in the induction of vascular permeability. Yet, since fungal loads were similar in the pICLC-dosed IFNar1 KO, IL-1r1 KO, and IL-6 KO mice, it was unclear if these cytokines were operating in the same pathway in the inhibition of Cg. To test this, mice deficient in both IL-1r1 and IFNar1 were infected with Cg and dosed with pICLC. These IL-1r1/IFNar1 double deficient mice showed an identical level of pICLC-mediated Cg inhibition loss (**Figure 6G**) compared to either IFNar1 KO or IL-1r1 KO strains (**Figures 6D, E**). While some vascular permeability independent inhibition of Cg remains in IFNar1 KO, IL-1r1 KO, and IL-6 KO mice, collectively these data show that substantial inhibition of Cg requires t1IFN, IL-1, and IL-6 and that t1IFN and IL-1 act in the same pathway to inhibit Cg infection.

### Vascular permeability inhibits Cg by iron restriction

**Figure 7:**
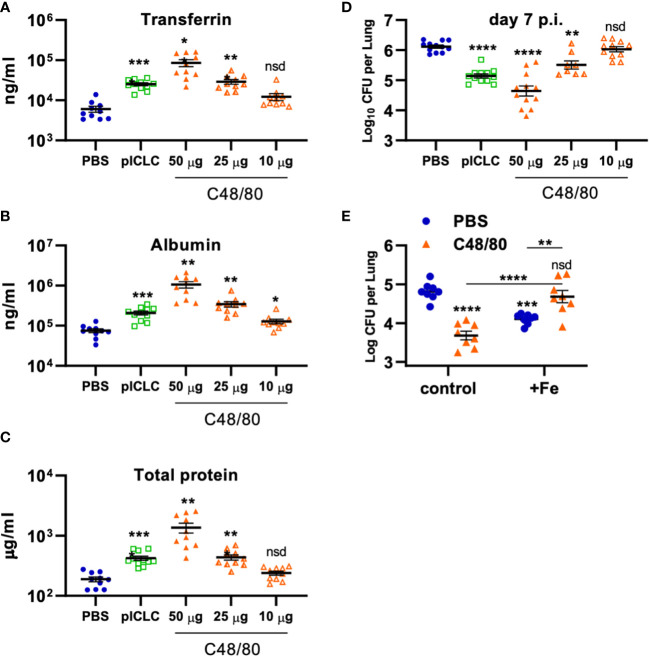
Vascular permeability induced by pICLC or compound 48/80 inhibits *C. gattii* in mice. **(A-C)** C57BL6 mice were dosed with PBS, 5 µg of pICLC, or indicated doses of compound 48/80. BAL samples were acquired after 3 days and vascular permeability marker levels measured by ELISA **(A, B)** or BCA **(C)**. See **Supplemental Figure 1C** for dosing and sampling schematic. N = 10-11 mice per group combined from 2 independent experiments. **(D)** C57BL6 mice were infected with Cg and dosed with PBS, 5 µg of pICLC, or indicated doses of compound 48/80. Lung fungal burdens were measured from lung homogenates after 7 days; see **Supplemental Figure 1D**. N = 8-12 mice per group combined from 3 independent experiments. For **(A-D)**, Statistics analyzed by one way Brown-Forsythe ANOVA and subsequent multiple comparison corrected t-tests compared to PBS control. **(E)** C57BL6 mice were dosed with PBS (Blue) or 50 µg compound 48/80 (orange) with or without additional 6.25 µg iron chloride the day before infection with Cg (6.25 µg iron chloride was included again in indicated groups). Compound 48/80 and iron chloride dosing was repeated on day 2 and lung fungal burdens were measured from lung homogenates 3 days after Cg infection. See **Supplemental Figure 1E** for dosing and sampling schematic. Statistics analyzed by two-way ANOVA and subsequent Tukey multiple comparison tests. **** indicates p < 0.0001, *** indicates p < 0.0002, ** indicates p < 0.0021, * indicates p < 0.0332, nsd indicates not statistically different.

To probe if Cg inhibition is directly mediated by vascular permeability, C48/80 was utilized to induce lung vascular permeability by a distinct mechanism of action from pICLC (25, 26). To compare the severity of lung vascular permeability induced by C48/80 to that of pICLC, BAL lung vascular permeability markers were measured from C48/80 or pICLC dosed mice. Samples from mice given C48/80 showed dose-dependent levels of Tfn, Alb, and total protein confirming that C48/80 induces lung vascular permeability (**Figures 7A–C**). Notably the middle dose of C48/80 (25 µg) induced very similar levels of lung vascular permeability markers as pICLC treatment. C48/80 also showed a dose dependent inhibition of Cg with very similar fungal loads in pICLC and the 25 µg c48/80 dose (**Figure 7D**). These data demonstrate a clear correlation between the extent of induced lung vascular permeability and Cg inhibition. Similar to pICLC (15), C48/80-induced Cg inhibition was also iron restriction dependent as addition of exogenous iron reversed Cg protection (**Figure 7E**). Thus, induced lung vascular permeability inhibits Cg infection.

## Discussion

We demonstrate that pICLC signaling through MDA5 induces both t1IFN and inflammatory factors including IL-1 and IL-6 and all these pathways are critical for the downstream induction of lung vascular permeability. While pICLC-induced lung vascular leakage was robustly measurable, this observed pICLC-induced permeability was moderate and did not seem associated with adverse pathology. This moderate-level vascular permeability is biologically significant as leakage delivers antimicrobial serum factors to lung airspaces which effectively inhibit Cg.

While IL-1 and IL-6 have recognized roles in vascular permeability (19, 31, 32), t1IFN has not been shown to be similarly involved. Our data clearly demonstrate that pICLC-induced lung vascular permeability entirely depends on t1IFN signaling (**Figure 4**). While poly(I:C) and MDA5 have previously been shown to induce some pro-inflammatory mediators (40), our data demonstrate a critical role for t1IFN in the induction of a broad pro-inflammatory response to double stranded RNA, including IL-1 and IL-6 (**Figures 4**, **5**). Although we acknowledge that these IL-10 protein induction data are less robust than those published previously by other groups, we demonstrate t1IFN-mediated expression of IL-1-inhibitory factors (IL-10, IL-1ra, Ch25h), in agreement with other data (34, 35, 39), suggesting that t1IFN both potentiates and suppresses IL-1 signaling in this model.

This leakage induced iron nutritional immunity is probably most effective against non-professional pathogens. Despite some early controversy, Cg is probably a non-professional opportunistic pathogen since many Cg-infected patients initially described as immunocompetent have subsequently demonstrated subtle immune deficiencies such as expression of cytokine neutralizing autoantibodies (12). These and previous data (15) support that Cg harvests Tfn iron inefficiently, further underlining Cg as an opportunistic pathogen. Thus, permeability-delivered iron binding proteins are restrictive to Cg [(15) and **Figures 6**, **7**]. Because vascular permeability is entirely dependent on IFNar1 (**Figures 4**, **6**) but pICLC-mediated protection from Cg was mostly but not entirely dependent on IFNar1 (**Figure 6**) these data also demonstrate the existence of vascular permeability independent mechanisms of pICLC-mediated resistance.

Many fungal lung pathogens are considered opportunistic and share iron acquisition strategies, and we and others have demonstrated pICLC-induced protection against several fungal species (16, 41). Thus, we speculate that MDA5-induced serum factor leakage may protect against other microbes, especially fungal opportunistic pathogens while we acknowledge other pICLC-responsive immune mechanisms are certainly involved (16, 41). For example, while we surmise that permeability-mediated iron restriction has some impact on *C. neoformans* infection, cellular immunity is much more important in this model than in Cg. Utility of serum leakage against secondary infection may not be limited to iron nutritional immunity as other immune factors were delivered, for example complement c3 (**Figure 1**) which has well-established anti-microbial functions (42). Inflammation, pICLC, and t1IFN all result in the recruitment of leukocytes (15, 16, 18). While these cells are likely to be important for combating other infections, pICLC-mediated protection from Cg infection has been shown to be independent of these recruited cells (15). Thus, when combined with the absence of overt host pathology these functional data support that MDA5-induction of limited vascular permeability is host beneficial suggesting a possible explanation for the evolutionary conservation of inflammatory induction of vascular leakage.

In contrast, serious infections and other damage can induce an uncontrolled and drastic level of vascular permeability resulting in a deadly cascade of pathology (20, 21, 43, 44). Comparing the low-level vascular permeability induced by pICLC-MDA5 to the extreme edema observed during some viral infections suggests that additional factors beyond MDA5 are involved in extreme lung vascular permeability. Additional pathogen PAMPS or DAMPS from host damage may modify or potentiate the induction of the pro-inflammatory signals or perturb the resolution of inflammation. Similarly, the induction of additional cytokines or chemokines beyond those studied here may contribute to the exacerbation of vascular permeability observed in some settings. Unlike some other fungal infections (45), Cg itself induces little inflammation and a muted cytokine response (46–48) and Cg infection does not alter vascular permeability levels (15). Additionally, many pathogens associated with ARDS inhibit t1IFN- and/or MDA5-signaling with specific virulence factors (28, 49) which could also contribute to loss of host control over these pathways. Prolonged or uncontrolled vascular permeability can also contribute to pathogenic fibrotic responses (50). We hope future studies will further illuminate the signaling perturbations which result in uncontrolled permeability and edema.

These data suggest that t1IFN and MDA5 pathway modulation may therapeutically benefit some ARDS cases. Since induced vascular permeability in this model was entirely t1IFN dependent (**Figure 5**) inhibiting t1IFN may be of therapeutic benefit in some ARDS patients although this may also be deleterious for anti-viral control. Additionally, many anti-inflammatory-type signals, including those that oppose the IL-1 pathway, are regulated by t1IFN [**Figure 7** and (34, 35, 39)] so t1IFN inhibition may detrimentally affect resolution pathways. Alternatively, these data support efforts to block proinflammatory pathways, such as IL-1 and IL-6, for controlling ARDS which have met with some success in COVID-19 (51, 52). Finally, nucleic acid sensing by MDA5 was sufficient to induce lung vascular permeability. Thus, the MDA5 and RIG-I common signaling pathway may also be an interesting target for therapeutic manipulation.

Overall, the data presented here demonstrate that MDA5-signaling induces both t1IFN and inflammatory cytokines. T1IFN expression enhances the pro-inflammatory cytokine signals by inducing positive feedback within the MDA5 pathway. This enhanced IL-1 and IL-6 expression mediates limited lung vascular permeability which delivers serum proteins to the lung airspace. Many of these serum proteins have innate immune function and protect the host lungs from microbial infection. Taken together, MDA5 sensing of viral RNA induces controlled vascular leakage which enhances innate immune defenses against opportunistic infection. These data demonstrate an evolutionary basis for the selection of immune control of vascular leakage and elucidate vascular permeability-regulating pathways which may be of therapeutic interest.

## Data availability statement

The raw data supporting the conclusions of this article will be made available by the authors, without undue reservation.

## Ethics statement

This study was reviewed and approved by The Institutional Animal Care and Use Committee of the National Institute of Allergy and Infectious Diseases (approval no. LCIM-5E).

## Author contributions

Conceived the study and participated in its design and coordination: MD, YC, KK-C. Carried out experiments and data analysis: MD, RM, GP, EH, SM. Wrote the manuscript: MD, YC, KK-C. Reviewed, edited, and/or provided critical scientific revision of the manuscript: MD, KM-B, YC, KK-C. All authors contributed to the article and approved the submitted version.

## Funding

This work was supported by a research fund from the intramural program of the National Institute of Allergy and Infectious Diseases, National Institutes of Health. The authors declare no competing financial interests.

## Acknowledgments

We wish to acknowledge Andres Salazar and Oncovir, Inc for the kind provision of poly(I:C)-LC, (hiltonol).

## Conflict of interest

The authors declare that the research was conducted in the absence of any commercial or financial relationships that could be construed as a potential conflict of interest.

## Publisher’s note

All claims expressed in this article are solely those of the authors and do not necessarily represent those of their affiliated organizations, or those of the publisher, the editors and the reviewers. Any product that may be evaluated in this article, or claim that may be made by its manufacturer, is not guaranteed or endorsed by the publisher.

## Abbreviations

ARDS, Acute Respiratory distress syndrome; Cg, *Cryptococcus gattii* outbreak strain R265; pICLC, Hiltonol, poly IC condensed with poly-L-lysine and carboxylcellulose; MDA5, melanoma differentiation-associated protein 5; c48/80, compound 48/80, mast cell degranulation reagent; t1IFN, type 1 interferon (IFN-β and many IFN-α’s); MDA5 genetic deletion mice, MDA5 KO; IFNar1 genetic deletion mice, IFNar1 KO; IL-1r1 genetic deletion mice, IL-1r1 KO; IL-6 genetic deletion mice, IL-6 KO; Transferrin, Tfn; Ferritin, Ftn; Albumin, Alb; bronchoalveolar lavage, BAL
