# Supplementary material for: MDA5 signaling induces type 1 IFN- and IL-1-dependent lung vascular permeability which protects mice from opportunistic fungal infection
Source: Front Immunol. 2022 Jul 28;13:931194. doi: 10.3389/fimmu.2022.931194 (PMC9368195; doi:10.3389/fimmu.2022.931194)
Supplement: Supplementary file 1 [file Presentation_1.pptx]

## Slide 1
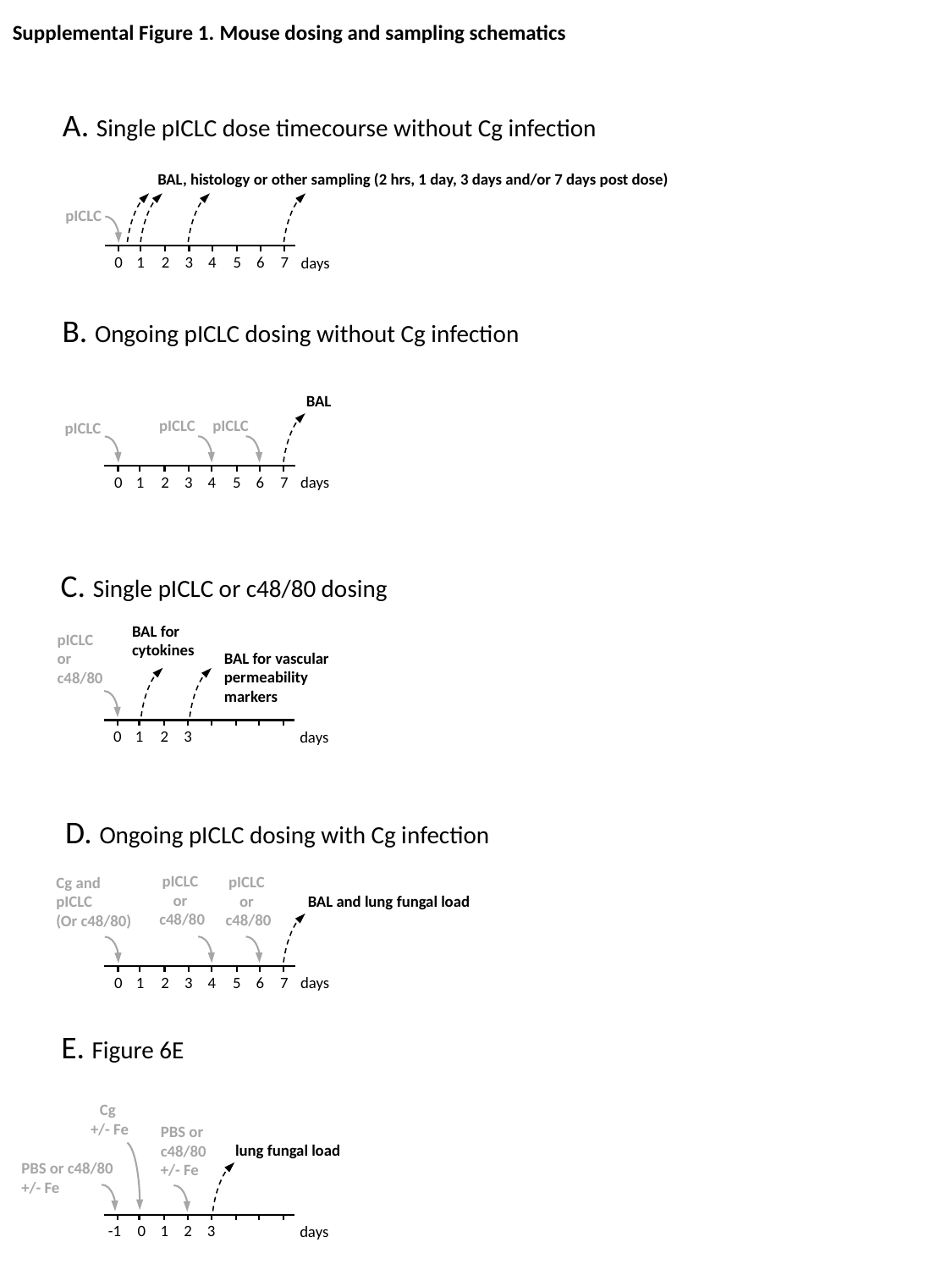

Supplemental Figure 1. Mouse dosing and sampling schematics
A. Single pICLC dose timecourse without Cg infection
BAL, histology or other sampling (2 hrs, 1 day, 3 days and/or 7 days post dose)
pICLC
0
1
2
3
4
5
6
7
days
B. Ongoing pICLC dosing without Cg infection
BAL
pICLC
pICLC
pICLC
0
1
2
3
4
5
6
7
days
C. Single pICLC or c48/80 dosing
BAL for cytokines
pICLC
or c48/80
BAL for vascular permeability markers
0
1
2
3
days
D. Ongoing pICLC dosing with Cg infection
pICLC
or
c48/80
pICLC
or
c48/80
Cg and
pICLC
(Or c48/80)
BAL and lung fungal load
0
1
2
3
4
5
6
7
days
E. Figure 6E
Cg
+/- Fe
PBS or
c48/80
+/- Fe
lung fungal load
PBS or c48/80
+/- Fe
-1
0
1
2
3
days

## Slide 2
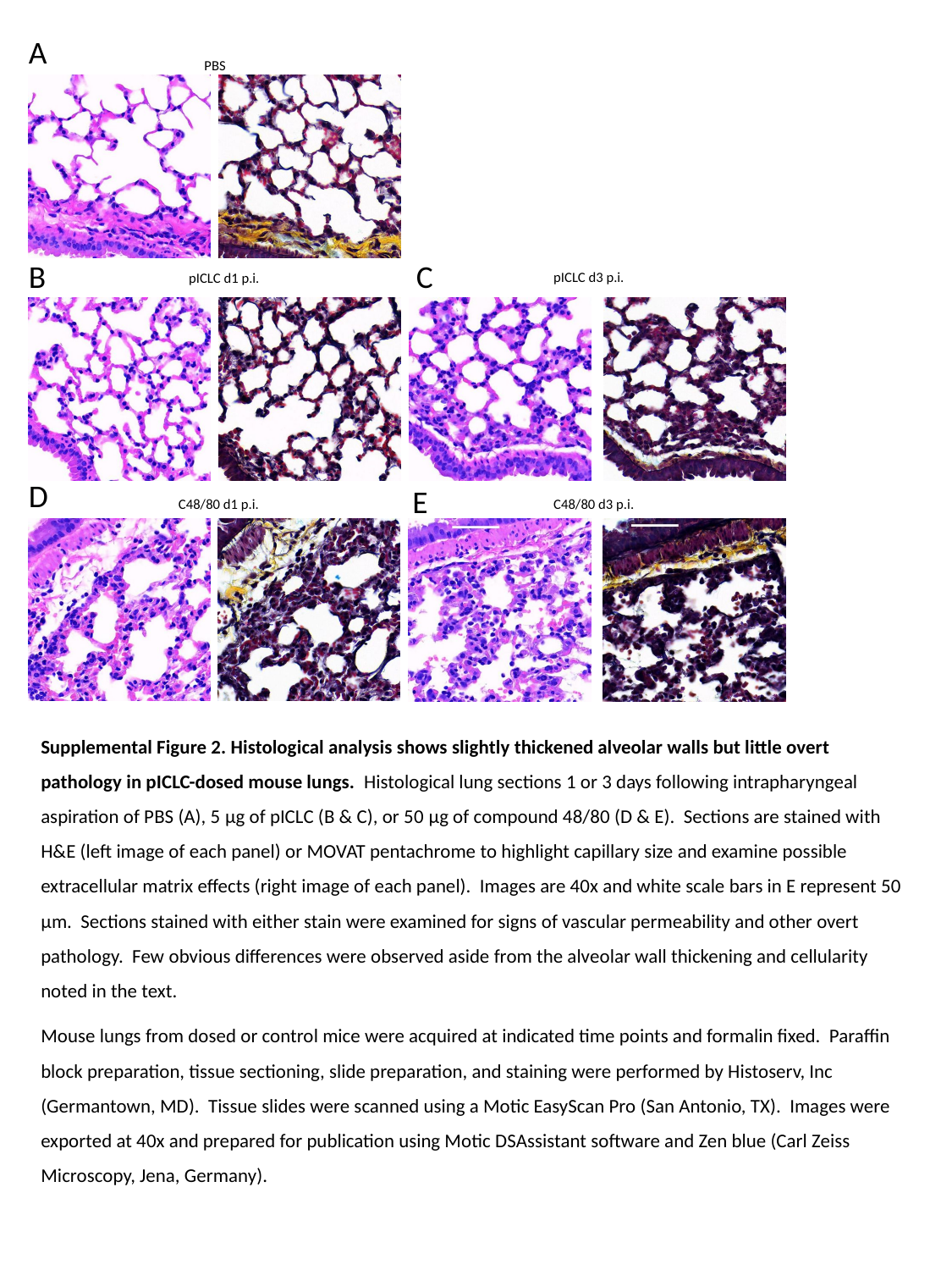

A
PBS
B
C
pICLC d3 p.i.
pICLC d1 p.i.
D
E
C48/80 d1 p.i.
C48/80 d3 p.i.
Supplemental Figure 2. Histological analysis shows slightly thickened alveolar walls but little overt pathology in pICLC-dosed mouse lungs. Histological lung sections 1 or 3 days following intrapharyngeal aspiration of PBS (A), 5 µg of pICLC (B & C), or 50 µg of compound 48/80 (D & E). Sections are stained with H&E (left image of each panel) or MOVAT pentachrome to highlight capillary size and examine possible extracellular matrix effects (right image of each panel). Images are 40x and white scale bars in E represent 50 µm. Sections stained with either stain were examined for signs of vascular permeability and other overt pathology. Few obvious differences were observed aside from the alveolar wall thickening and cellularity noted in the text.
Mouse lungs from dosed or control mice were acquired at indicated time points and formalin fixed. Paraffin block preparation, tissue sectioning, slide preparation, and staining were performed by Histoserv, Inc (Germantown, MD). Tissue slides were scanned using a Motic EasyScan Pro (San Antonio, TX). Images were exported at 40x and prepared for publication using Motic DSAssistant software and Zen blue (Carl Zeiss Microscopy, Jena, Germany).

## Slide 3
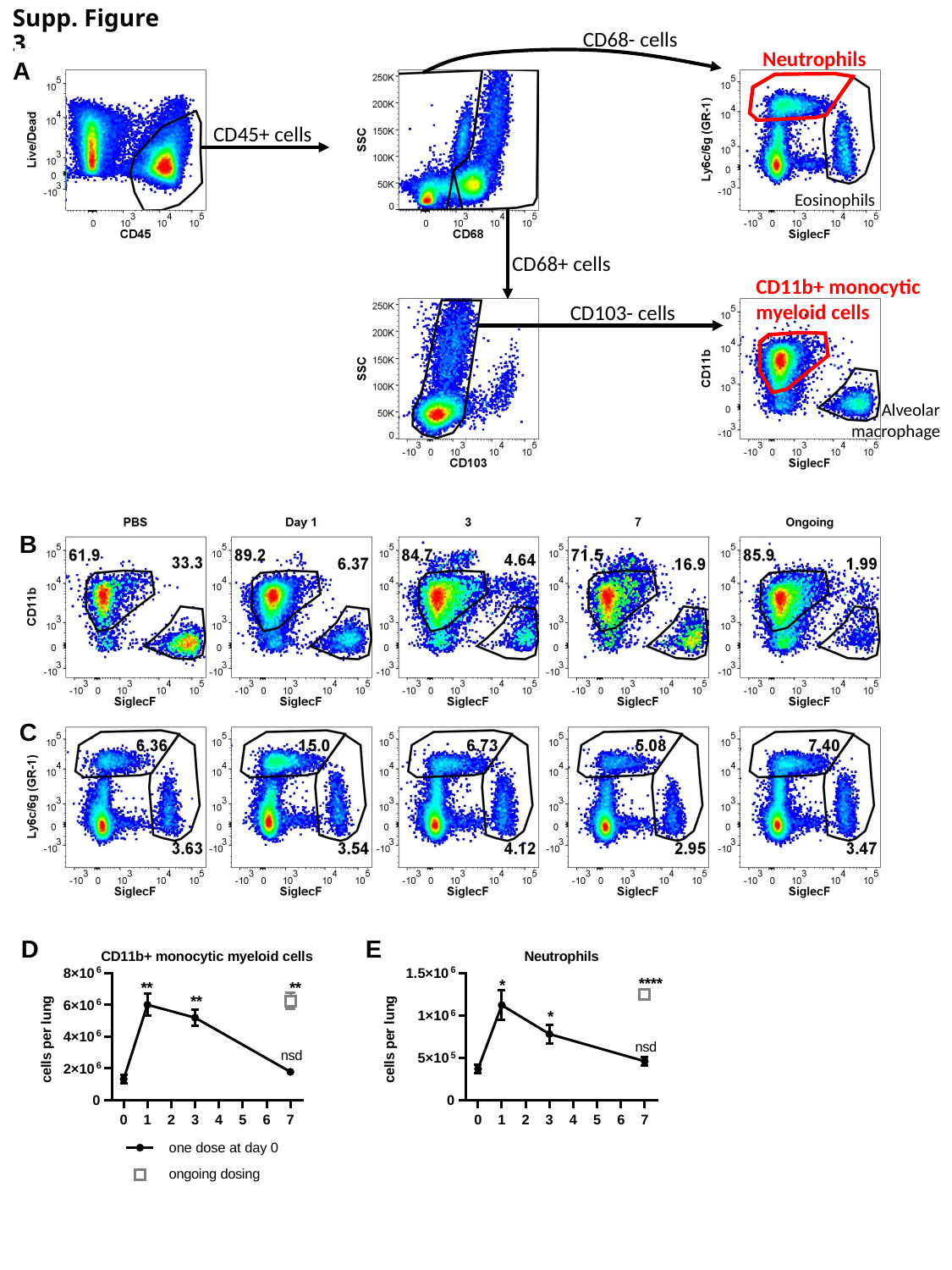

Supp. Figure 3.
CD68- cells
Neutrophils
A
CD45+ cells
Eosinophils
CD68+ cells
CD11b+ monocytic myeloid cells
CD103- cells
Alveolar macrophage
B
C

## Slide 4
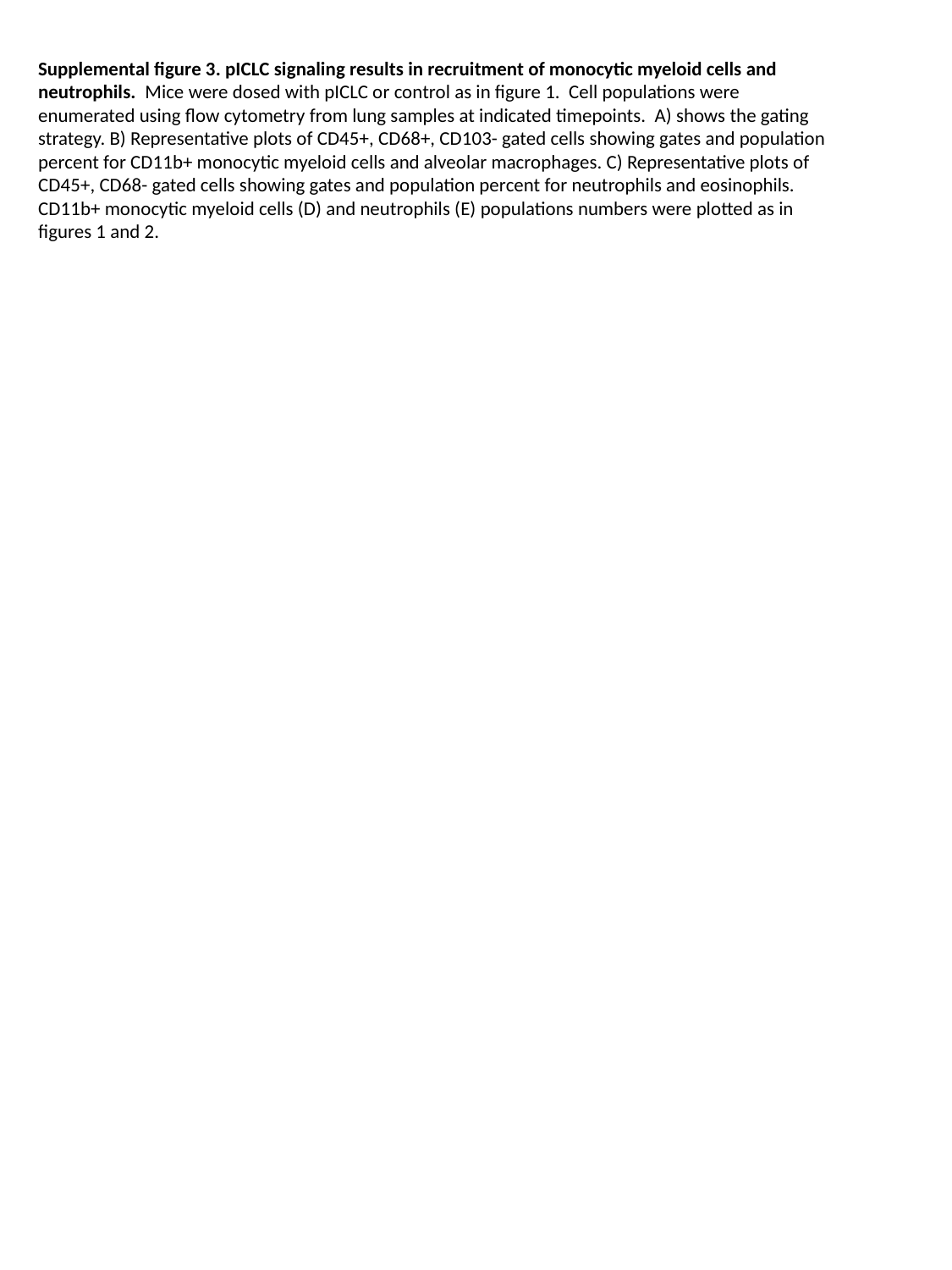

Supplemental figure 3. pICLC signaling results in recruitment of monocytic myeloid cells and neutrophils. Mice were dosed with pICLC or control as in figure 1. Cell populations were enumerated using flow cytometry from lung samples at indicated timepoints. A) shows the gating strategy. B) Representative plots of CD45+, CD68+, CD103- gated cells showing gates and population percent for CD11b+ monocytic myeloid cells and alveolar macrophages. C) Representative plots of CD45+, CD68- gated cells showing gates and population percent for neutrophils and eosinophils. CD11b+ monocytic myeloid cells (D) and neutrophils (E) populations numbers were plotted as in figures 1 and 2.

## Slide 5
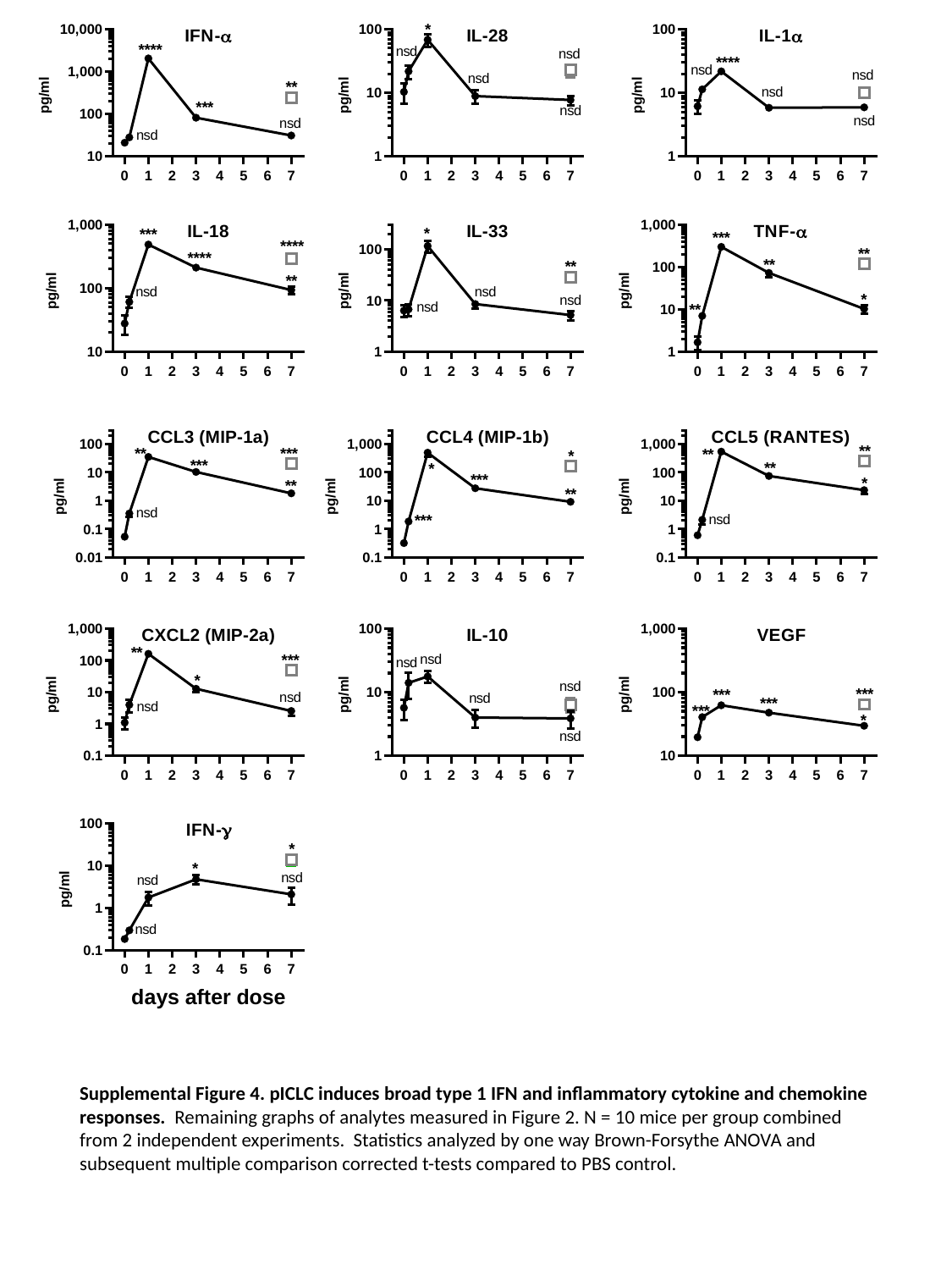

Supplemental Figure 4. pICLC induces broad type 1 IFN and inflammatory cytokine and chemokine responses. Remaining graphs of analytes measured in Figure 2. N = 10 mice per group combined from 2 independent experiments. Statistics analyzed by one way Brown-Forsythe ANOVA and subsequent multiple comparison corrected t-tests compared to PBS control.

## Slide 6
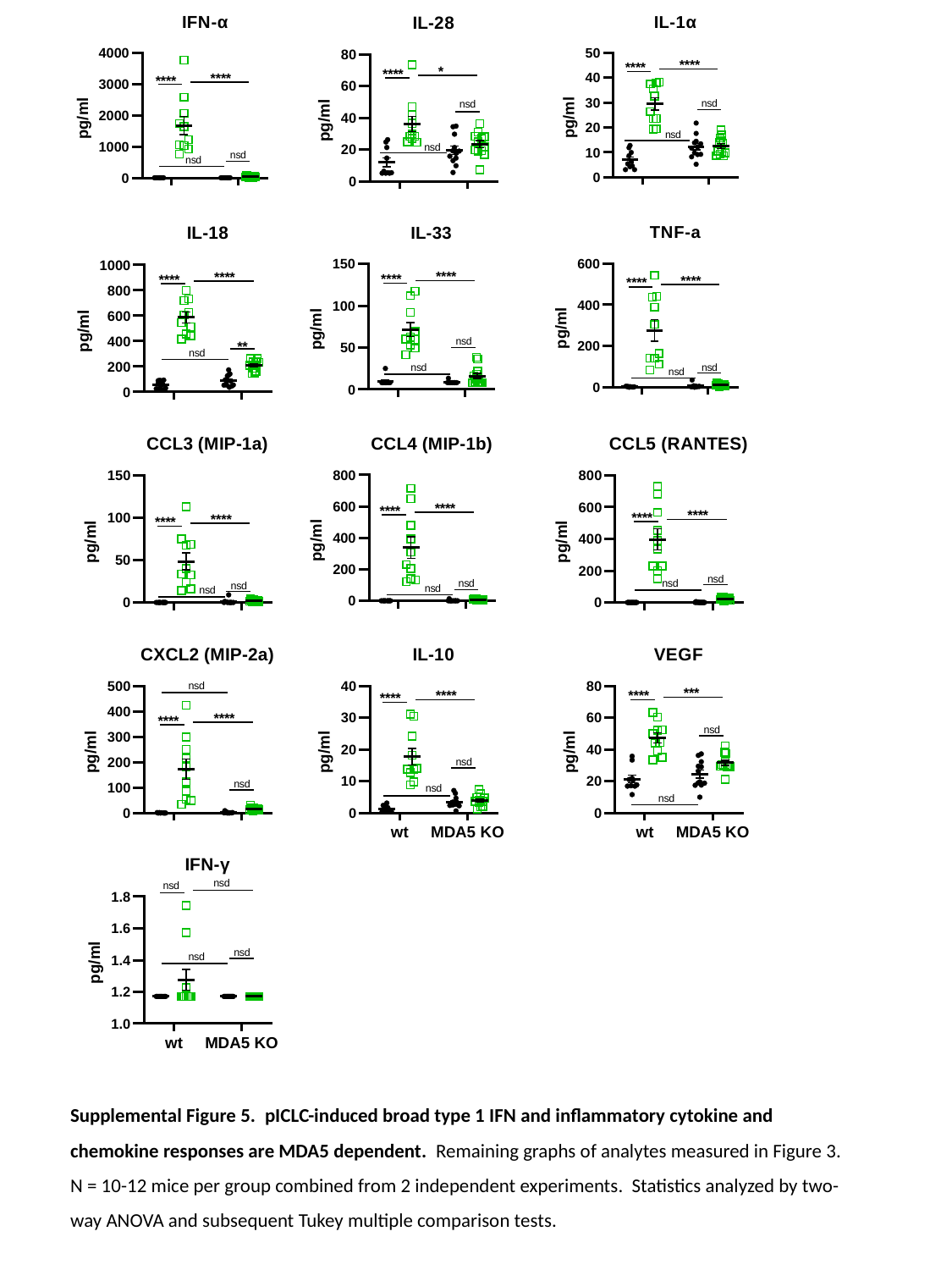

Supplemental Figure 5. pICLC-induced broad type 1 IFN and inflammatory cytokine and chemokine responses are MDA5 dependent. Remaining graphs of analytes measured in Figure 3. N = 10-12 mice per group combined from 2 independent experiments. Statistics analyzed by two-way ANOVA and subsequent Tukey multiple comparison tests.

## Slide 7
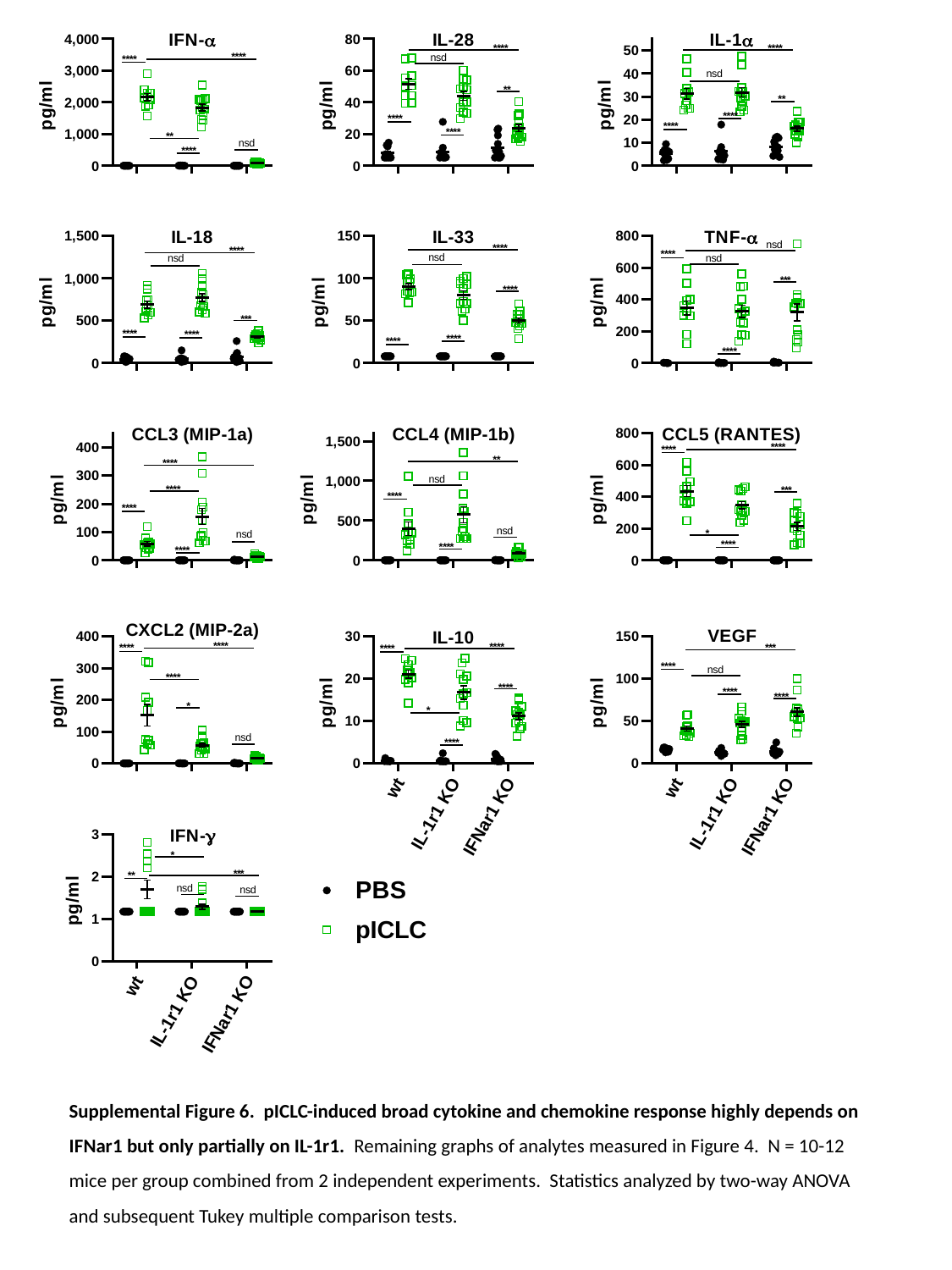

Supplemental Figure 6. pICLC-induced broad cytokine and chemokine response highly depends on IFNar1 but only partially on IL-1r1. Remaining graphs of analytes measured in Figure 4. N = 10-12 mice per group combined from 2 independent experiments. Statistics analyzed by two-way ANOVA and subsequent Tukey multiple comparison tests.
